# Intercostal Artery Screening with Color Doppler Thoracic Ultrasound in Pleural Procedures: A Potential Yet Underexplored Imaging Modality for Minimizing Iatrogenic Bleeding Risk in Interventional Pulmonology

**DOI:** 10.3390/jcm14176326

**Published:** 2025-09-07

**Authors:** Guido Marchi, Sara Cinquini, Francesco Tannura, Giacomo Guglielmi, Riccardo Gelli, Luca Pantano, Giovanni Cenerini, Valerie Wandael, Beatrice Vivaldi, Natascia Coltelli, Giulia Martinelli, Alessandra Celi, Salvatore Claudio Fanni, Massimiliano Serradori, Marco Gherardi, Luciano Gabbrielli, Francesco Pistelli, Laura Carrozzi

**Affiliations:** 1 Pulmonology Unit, Cardiothoracic and Vascular Department, University Hospital of Pisa, 56124 Pisa, Italy; 2 Department of Surgical, Medical and Molecular Pathology and Critical Care Medicine, University of Pisa, 56126 Pisa, Italy; 3 Academic Radiology, Department of Translational Research, University of Pisa, 56126 Pisa, Italy

**Keywords:** intercostal artery (ICA), color Doppler ultrasound (CDUS), thoracic ultrasound (TUS), pleural procedures, pleural diseases, thoracentesis, chest tube, hemorrhagic complications, ultrasound-guided interventions, artificial intelligence (AI)

## Abstract

Hemorrhagic complications during pleural interventions—such as thoracentesis and chest tube insertion—remain a significant clinical concern, primarily due to inadvertent injury of the intercostal artery (ICA). The highly variable ICA anatomy is frequently not visualized on conventional imaging, limiting the reliability of landmark-based techniques. Color Doppler thoracic ultrasound (CDUS) has emerged as a non-invasive, real-time modality capable of identifying ICAs and their anatomical variants prior to pleural access. This narrative review synthesizes current evidence on CDUS-guided ICA screening, focusing on its technical principles, diagnostic performance, and clinical applicability. While feasibility and utility are supported by multiple observational studies, robust evidence demonstrating a reduction in bleeding complications is still lacking. Barriers to widespread implementation include heterogeneous scanning protocols, operator dependency, and the absence of standardized training. We discuss the anatomical rationale for pre-procedural vascular mapping and highlight emerging protocols aimed at standardizing ICA visualization. Although not yet incorporated into major clinical guidelines, CDUS represents a promising tool to enhance procedural safety. Emerging AI applications may further improve vessel detection by reducing operator dependency and enhancing reproducibility. High-quality prospective studies are essential to validate potential clinical benefits, optimize implementation strategies, and support integration into routine pleural practice.

## 1. Introduction

Pleural procedures such as thoracentesis, intercostal chest drain (ICD) insertion, indwelling pleural catheter (IPC) placement, ultrasound-guided pleural biopsy, and medical thoracoscopy are cornerstone interventions in respiratory medicine, valued for their diagnostic and therapeutic utility [1]. As the global burden of pleural diseases rises and dedicated pleural services continue to expand worldwide, the volume of these procedures performed by physicians continues to grow [2,3]. Despite their routine nature and generally favorable safety profile, pleural interventions are not without risk [4,5]. Among the most serious complications is iatrogenic vascular injury, particularly involving the intercostal arteries (ICAs), which can result in hemothorax, hemodynamic instability, or even death [6,7]. Although infrequent, such bleeding events can be life-threatening due to the unique anatomy of the pleural space, where negative intrathoracic pressure and the absence of effective external compression limit hemostatic control [8,9]. Reported rates of hemorrhage reach up to 2% for thoracentesis, 13% for ICD insertion, and 4% for thoracoscopy, though under-reporting likely underestimates the true incidence [10]. This risk is further accentuated in patients with coagulopathies or under antithrombotic treatment, where even minor arterial trauma may lead to disproportionate clinical consequences. Operator experience and standard thoracic ultrasound (TUS) reduce complications such as pneumothorax or visceral injury but offer no protection against ICA laceration [11]. The ability of color Doppler ultrasound (CDUS) to visualize ICAs and their collaterals in real time has been demonstrated and supported in initial studies [12,13,14]. However, its role remains underexplored in real-world clinical populations and is still absent from major procedural guidelines [11,15]. Traditional reliance on anatomical landmarks and static imaging falls short in addressing the variability and unpredictability of ICA anatomy, even in presumed “safe” zones. In this context, CDUS has emerged as a promising, non-invasive modality capable of individualized vascular mapping prior to pleural access. This review synthesizes the current evidence on CDUS-based ICA screening, examining its anatomical rationale, diagnostic performance, and barriers to clinical adoption, with the aim of informing future research and advancing procedural safety.

## 2. Methods

This narrative review was conducted through a comprehensive literature search aimed at identifying studies investigating the visualization and mapping of ICAs using CDUS, as well as its clinical utility in preventing hemorrhagic complications during pleural procedures. The search included major biomedical databases—PubMed, Embase, Web of Science, Google Scholar, and Scopus—covering publications up to July 2025.

The search terms encompassed, but were not limited to, “intercostal artery,” “pleural procedures,” “thoracentesis”, “thoracic ultrasound,” “color Doppler,” “bleeding risk,” “vascular injury,” “ultrasound-guided intervention,” “hemothorax”, and “hemorrhagic complications.”

We focused on original research articles, clinical trials, observational studies, and case series involving human subjects published in English. Conference abstracts, editorials, and studies unrelated to the topic were not included.

Articles were reviewed for relevance, and information was gathered on study design, population characteristics, ultrasound methodology (including probe types and scanning protocols), clinical outcomes (particularly hemorrhagic complication rates), and reported limitations. Given the heterogeneity of available studies, a narrative synthesis approach was adopted.

The objective was to provide a clinically relevant, evidence-based overview of current knowledge, elucidate the potential benefits and challenges of CDUS screening of ICAs, and identify gaps warranting further research.

## 3. Anatomical Overview of Intercostal Arteries: Variations and Clinical Implications

### 3.1. Gross Anatomy and Common Variants of the Intercostal Arteries

**Figure 1:**
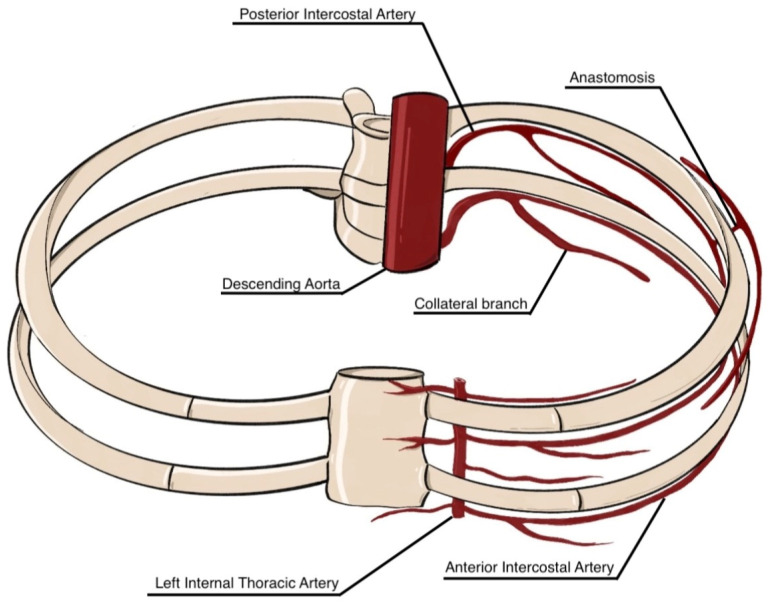
Anatomical representation of the intercostal arterial system. The posterior intercostal arteries arise from the thoracic (descending) aorta, while the anterior intercostal arteries originate from the internal thoracic artery. Collateral branches and anastomoses between anterior and posterior intercostal arteries ensure segmental perfusion of the thoracic wall (this image is a schematic representation for illustrative purposes only. It is not intended to depict the exact anatomical reality but rather to highlight key concepts in a simplified manner).

The ICAs are vital for thoracic wall perfusion, particularly the intercostal muscles involved in respiration. These vessels are categorized into anterior and posterior ICAs [16]. Each intercostal space receives blood from one posterior and two anterior ICAs, providing robust collateral circulation [16,17] (Figure 1).

The posterior intercostal arteries (PIAs) are of particular clinical relevance owing to their anatomical course and location, which make them vulnerable to iatrogenic injury during pleural procedures, such as thoracentesis or tube thoracostomy [18]. The first and second PIAs arise from the supreme (or superior) ICA, a branch of the costocervical trunk from the subclavian artery, whereas PIAs from the third to the eleventh intercostal space derive directly from the descending thoracic aorta. The aortic origin, left of midline, imposes a longer, oblique path on right-sided PIAs [16].

Each PIA travels within the neurovascular bundle beneath the corresponding rib, arranged superior to inferior as vein, artery, and nerve, housed in the costal groove between the internal and innermost intercostal muscles. As it traverses the intercostal space, the PIA anastomoses with anterior ICAs derived from either the internal thoracic artery (ITA) or the musculophrenic artery and gives rise to dorsal, muscular, collateral, and cutaneous branches [16,19,20].

Anterior intercostal arteries (AIAs) in the upper six intercostal spaces originate from the ITA, a subclavian branch descending anterior to the pleura, which also gives rise to pericardiophrenic, sternal, and mediastinal branches. Beyond the sixth space, AIAs originate from the musculophrenic artery, one of the terminal branches of the ITA, which bifurcates around the sixth intercostal space into the musculophrenic and superior epigastric arteries [21,22,23].

Despite this canonical pattern, significant anatomical variation exists. Table 1 summarizes the findings from cadaveric dissections and virtual 3D models [24], which are increasingly employed in medical education. However, autopsy studies remain indispensable for elucidating human vascular variability and informing surgical practice.

**Figure 2:**
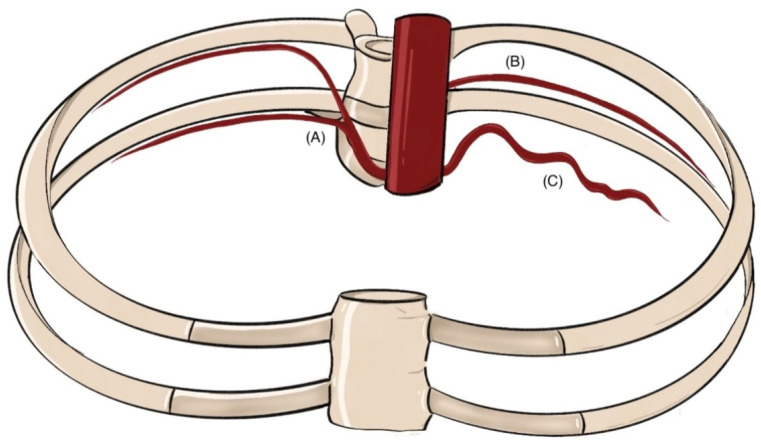
Three representative anatomical variants in the course of the intercostal artery (ICA) are illustrated: (**A**) the typical pattern, in which the ICA runs along the inferior margin of the rib within the costal groove (subcostal location); (**B**) a high-positioned ICA coursing closer to the midportion of the intercostal space; and (**C**) an aberrant or tortuous ICA traversing centrally through the intercostal space (this image is a schematic representation for illustrative purposes only. It is not intended to depict the exact anatomical reality but rather to highlight key concepts in a simplified manner).

Variations affect the PIAs’ origin, number, caliber, and trajectory (Figure 2). Typically, PIA ostia appear on the medial aspect of the aortic posterior wall in upper thoracic segments, rotating dorsally at lumbar levels. Axial spacing is generally wider in the thorax, and longitudinal distance increases inferiorly, though positional deviations are common [26,28].

Absence of one or more PIA ostia may be congenital or acquired, e.g., due to atherosclerosis [28], but thoracic perfusion is preserved via a dense collateral and anastomotic network [32].

A frequent variation involves common trunks (CTs), where two or more ipsilateral PIAs share a single aortic origin. Kocbek et al. observed at least one CT in 70% of 44 cadavers, predominantly in upper intercostal spaces; up to four CTs were documented, with a rare case describing five [29]. CT formation likely stems from incomplete fusion of embryonic intersegmental arteries [33].

In rare cases, PIAs follow a dorsal trajectory through the costotransverse foramina, termed thoracic vertebral arteries, reported exclusively in the first four PIA pairs, all arising via CTs [29].

Significant tortuosity, especially in the elderly, can hinder procedural access. This, coupled with extensive branching, necessitates vigilance during thoracic interventions [30].

### 3.2. Radiological Assessment of Intercostal Artery Anatomy

Radiological imaging is pivotal in evaluating ICAs, particularly in pre-procedural planning and managing thoracic vascular complications. Contrast-enhanced computed tomography (CECT), and more specifically CT angiography (CTA), offers detailed, multiplanar assessment of ICAs and their branches [18].

Although ICAs maintain a segmental and typically predictable distribution [34], their course, origin, and caliber may exhibit marked individual variability, underscoring the importance of imaging in high-risk interventions.

**Figure 3:**
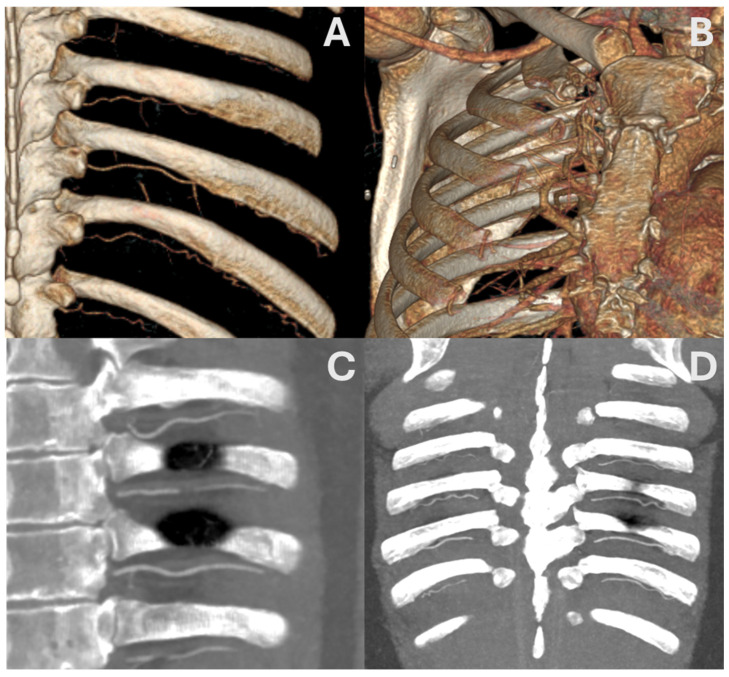
Contrast-enhanced CT acquired at the arterial phase. Volume rendering reconstruction showing left (**A**) and right (**B**) posterior intercostal arteries. Oblique reformatted image with maximum-intensity-projection reconstruction showing left posterior intercostal arteries (**C**). Coronal reformatted image with maximum-intensity-projection reconstruction showing both right and left posterior intercostal arteries (**D**).

CTA remains the modality of choice, enabling reliable mapping of vessel trajectory and detecting anatomical variants or tortuosity—especially relevant in older individuals or in more cephalad intercostal spaces, where deviations from classical anatomy are more frequent [35] (Figure 3). Notably, CTA demonstrates that ICAs situated >6 cm lateral to the spinous processes are shielded by the superior rib, reducing the risk of vascular injury during pleural access [35].

Although MRI is suboptimal for small-caliber vessels, high-resolution MR angiography allows identification of the artery of Adamkiewicz, a major radiculomedullary branch—typically arising between T8 and L2—of relevance in planning thoracoabdominal aortic repair [36].

**Figure 4:**
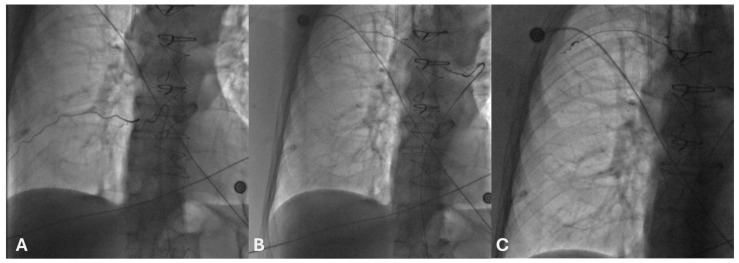
Digital subtraction angiography images of the right posterior intercostal arteries at the 8th (**A**) and 6th (**B**) intercostal spaces following selective catheterization. Embolization of the right 6th posterior intercostal artery was performed using non-magnetic coils (**C**).

Digital subtraction angiography (DSA) is the gold standard for the precise assessment and treatment of ICA pathology (Figure 4). DSA offers dynamic, high-resolution imaging with real-time catheter navigation, enabling selective visualization of small-caliber ICAs and their branches. This is particularly valuable in cases of active hemorrhage, where pinpoint localization of contrast extravasation facilitates targeted embolization [18] (Figure 4).

From a historical radiological perspective, CDUS has demonstrated the ability to occasionally visualize ICAs, especially along the lateral thoracic wall or when vessels are dilated; however, its sensitivity is variable and highly operator-dependent [13], and as such, it is considered a complementary tool rather than a substitute for CT-based vascular mapping.

## 4. Pleural Diseases and Interventional Procedures: Clinical Context and Techniques

### 4.1. Epidemiology and Clinical Spectrum of Pleural Diseases

Pleural diseases encompass a heterogeneous group of conditions, frequently associated with the development of pleural effusions (PEs) [37]. They are broadly classified into neoplastic disorders—such as primary pleural malignancies (e.g., mesothelioma) and secondary metastatic involvement—and non-neoplastic conditions, including pleural infections, fibrotic thickening, asbestos-related plaques, and pneumothorax [38]. These entities vary in clinical presentation, ranging from asymptomatic incidental findings to debilitating symptoms such as dyspnea, chest pain, and cough [37].

Pleural pathologies are associated with considerable morbidity and mortality, often impairing quality of life and necessitating urgent diagnostic and therapeutic interventions [39,40]. They account for a significant portion of emergency department visits and hospitalizations. In the United States, over 350,000 admissions annually are attributable to pleural diseases, with global incidence on the rise [41,42]. Consequently, they represent a growing clinical and economic burden due to escalating costs of evaluation, treatment, and follow-up [41,42].

In response to this increasing burden, dedicated pleural services are being established worldwide within respiratory medicine departments [2]. These multidisciplinary settings integrate interventional pulmonology techniques to address both diagnostic and therapeutic needs across a broad range of pleural conditions, including unexplained effusions, malignant pleural involvement, infections, pneumothorax, and hemothorax. The implementation of such services reflects a shift toward more structured, minimally invasive, and patient-centered care models [15].

### 4.2. Overview of Interventional Pulmonology Techniques

A range of minimally invasive procedures is now routinely employed within pleural services.

**Figure 5:**
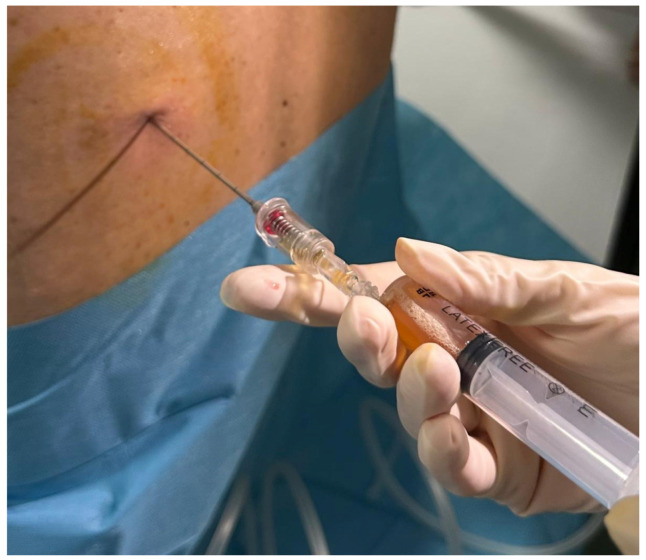
Ultrasound-guided diagnostic thoracentesis in a patient with pleural effusion. The image shows needle insertion into the posterior hemithorax, with aspiration of a straw-colored pleural fluid sample. The procedure is performed under aseptic conditions and local anesthesia, using negative pressure to collect fluid for cytological, biochemical, and microbiological analysis.

Thoracentesis (Figure 5) remains the most commonly performed pleural procedure and is essential in the initial evaluation of effusions. It allows biochemical, cytological, and microbiological analysis and relieves dyspnea. Ultrasound guidance is standard, significantly lowering complication rates, including pneumothorax [43,44,45].

Chest tube insertion is indicated for evacuating air or fluid in pneumothorax, hemothorax, empyema, or large effusions causing respiratory compromise. Catheter size (small-, medium-, or large-bore) is selected based on clinical context [46,47,48].

Image-guided pleural biopsy, under ultrasound or CT, is a minimally invasive, high-yield technique for sampling pleural thickening, nodules, or non-diagnostic effusions. It is crucial when cytology is inconclusive, enabling histopathologic and molecular characterization [49,50,51,52].

Medical thoracoscopy provides direct pleural visualization for biopsy and therapeutic procedures such as talc poudrage. Performed under local anesthesia with sedation, it offers high diagnostic accuracy and low invasiveness, and it is central to evaluating undiagnosed exudative effusions [53,54,55,56,57].

Indwelling pleural catheters (IPCs) offer outpatient palliation for recurrent malignant effusions, especially in trapped lung or failed/contraindicated pleurodesis [58,59]. IPCs reduce admissions and support patient-managed drainage at home [57,60].

Pleurodesis, aiming to obliterate the pleural space and prevent recurrence of effusions or pneumothorax, is performed by instilling sclerosing agents (e.g., talc) either via chest tube (slurry) or during thoracoscopy (poudrage). Lung re-expansion and patient selection are key to success [61,62,63].

Collectively, these interventional techniques constitute the cornerstone of modern pleural disease management in pulmonology. Their minimally invasive approach, flexibility across care settings, and strong diagnostic and therapeutic impact make them indispensable to evidence-based, patient-centered respiratory care.

## 5. Assessment of Hemorrhagic Risk and Strategies for Prevention in Pleural Interventions

### 5.1. Overview of Common Complications Associated with Pleural Procedures

Pleural interventions are essential in thoracic medicine but carry inherent risks [64,65,66]. The most frequent complication is iatrogenic pneumothorax, historically observed in up to 18% of procedures without ultrasound guidance. With routine ultrasound use—now the standard of care—this rate decreases to below 1.5% [5,67]. Infectious complications, including wound-site cellulitis and empyema, though less common, remain clinically significant, especially with IPCs, with reported incidence under 5% [5,68,69]. Direct injury to adjacent organs (e.g., liver, spleen, diaphragm) is now rare (<1%), largely prevented by real-time imaging [9]. Other adverse effects include procedural pain, vasovagal episodes, and, more rarely, re-expansion pulmonary edema—a potentially serious event [5].

**Figure 6:**
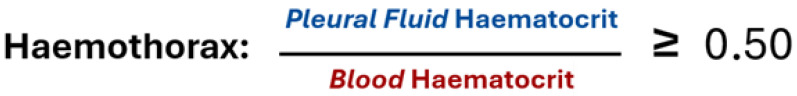
Diagnostic criterion for hemothorax based on the pleural fluid-to-blood hematocrit ratio. A hemothorax is defined when the pleural fluid hematocrit is ≥50% of the peripheral blood hematocrit.

Although less frequent than pneumothorax, hemorrhagic complications are the most acutely life-threatening [7]. Bleeding may range from minor vessel injury to massive hemothorax [70]. The latter is defined as a pleural fluid hematocrit ≥50% of peripheral blood, a threshold distinguishing true hemothorax from serosanguinous effusions and guiding clinical assessment [71,72,73] (Figure 6).

### 5.2. Incidence and Severity of Hemorrhagic Complications in Pleural Interventions

Clinically significant hemorrhage is a rare but serious complication of pleural procedures. Thoracentesis carries a low bleeding risk; a large 12-year retrospective study of over 9000 procedures reported complications under 1% [74]. Even in high-risk patients—such as those with uncorrected coagulopathies or on antithrombotic therapy—recent evidence indicates bleeding risk remains low when ultrasound guidance is strictly applied [75,76], challenging previous contraindications.

For ICD insertion, hemorrhagic risk varies by catheter size, technique, and clinical context [46,48]. Small-bore tubes placed under image guidance with the Seldinger technique are considered to be associated with lower bleeding risk, whereas large-bore catheters inserted by blunt dissection, especially in trauma contexts, are presumed to have higher complication rates, though comparative studies remain limited [5].

Ultrasound-guided pleural biopsies show a favorable safety profile; older blind methods had bleeding rates around 2%, while image-guided approaches reduce major hemorrhagic events to ~1% [52]. No significant bleeding difference exists between cryobiopsy and forceps biopsy [77].

Bleeding with IPCs is uncommon, usually minor, and rarely requires intervention. Medical thoracoscopy carries low overall bleeding risk, which may increase during extensive biopsy [68,78].

**Figure 7:**
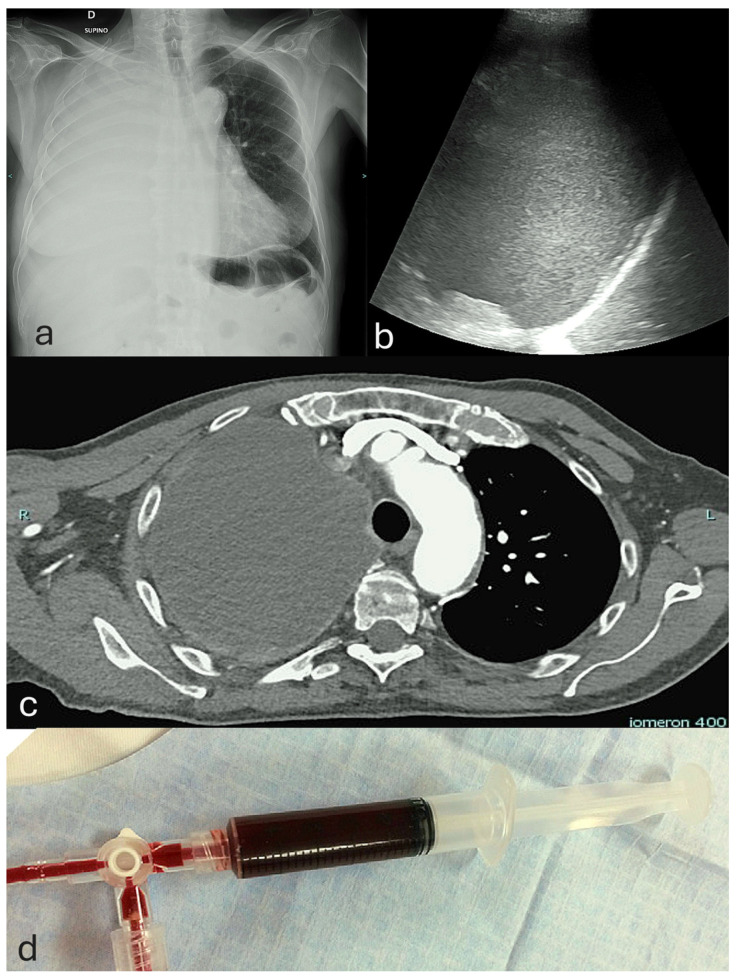
Multimodal imaging of hemothorax: (**a**) Chest X-ray (supine view) showing homogeneous opacification of the right hemithorax due to a large pleural effusion with contralateral mediastinal shift. (**b**) Thoracic ultrasound with a convex probe demonstrating a homogeneously echogenic pleural effusion suggestive of hemothorax in the acute or early subacute phase, reflecting the high cellularity of blood and resulting in marked acoustic reflection. (**c**) Contrast-enhanced chest computed tomography confirming the presence of hyperdense pleural fluid, consistent with hemothorax. (**d**) Pleural fluid aspiration yielding frankly hemorrhagic fluid. Hemothorax was definitively confirmed by a pleural fluid-to-blood hematocrit ratio ≥ 50% of the peripheral blood hematocrit.

ICA injuries, though rare, can cause life-threatening hemothorax due to the pleural space’s negative pressure and lack of tamponade, often necessitating surgery and increasing morbidity and healthcare burden [5,68,76] (Figure 7).

Hemothorax severity was traditionally classified by blood volume, but this static model may not reflect clinical complexity. A physiology-based approach, focusing on hemodynamics and bleeding rate, is favored. Hemodynamically stable patients without active bleeding can be managed conservatively [79], while progressive hemorrhage—initial ICD output > 1000–1500 mL or ongoing losses > 200 mL/h—often requires surgical control via thoracotomy or VATS [79,80]. Retained hemothorax, defined by incomplete drainage rather than volume alone, increases risk for late complications such as empyema and fibrothorax, requiring fibrinolysis or decortication [81,82,83,84,85].

### 5.3. Procedural Safety Measures and Risk Mitigation for Hemorrhagic Complications in Pleural Interventions

Pleural procedures require structured pre-procedural planning to ensure patient safety and minimize complications. International recommendations from BTS, ERS, ATS, and ACCP emphasize performing interventions in adequately equipped settings, using sterile technique and proper monitoring, and with availability of resuscitation equipment [15,69,86,87]. Operators must confirm laterality, obtain informed consent, and ensure correct patient positioning. Patient evaluation should include coagulation status, with correction advised to achieve INR ≤ 1.5 and platelet count ≥ 50,000/µL prior to non-urgent procedures [9]. Although traditionally considered essential, recent evidence indicates mild-to-moderate coagulopathy may not significantly increase bleeding risk when ultrasound guidance is used [74,75,88]. Antithrombotic agents, including warfarin, DOACs, and P2Y12 inhibitors, should be withheld per established protocols to balance thrombosis and bleeding risk [89,90]. Complex coagulopathies from hepatic dysfunction or DIC pose higher hemorrhagic risk and warrant correction and specialist consultation [88].

Ultrasound guidance is strongly recommended for all pleural interventions, both for site localization and real-time needle visualization, as it significantly reduces complication rates [5,11,88]. The use of small-bore catheters (≤14 Fr), proper anatomical landmarks such as the “safe triangle,” and correct needle orientation (just above the rib) are further safety measures endorsed to limit the risk of organ or vascular injury [12,15]. Adherence to these comprehensive measures results in very low rates of major bleeding, even in patients with anticoagulation or moderate coagulopathy [75,76].

Among the various measures aimed at minimizing bleeding risk, the pre-procedural use of CDUS to screen for ICAs represents a promising area of investigation and warrants critical examination of the available evidence supporting its role in pleural procedures.

## 6. Principles and Techniques of Color Doppler Thoracic Ultrasound Imaging

### 6.1. Principles of Color Doppler Imaging in Thoracic Ultrasound

CDUS represents an evolution of conventional B-mode ultrasonography, enabling real-time visualization and qualitative assessment of blood flow by analyzing interactions between acoustic pulses and moving erythrocytes within vascular structures [91,92]. Fast-moving intravascular elements generate distinct echo signals, processed into color flow maps [93]. These are conventionally displayed using the “BART” color-coding system—Blue Away, Red Towards—indicating flow direction relative to the ultrasound probe [91,92].

When blood flow is visualized as a waveform rather than a color map, the modality is called spectral Doppler, allowing semi-quantitative analysis of flow parameters such as velocity, pulsatility, and resistance indices [94].

Power Doppler (PD), a variant of CDUS, displays the amplitude (power) of the Doppler signal rather than frequency shift [95]. Although lacking directional information, PD is angle-independent and more sensitive to low-velocity flow, making it valuable for detecting small vessels or flow in deeper tissues [95].

In TUS, PD facilitates reliable identification of ICAs and other thoracic vessels by enhancing visibility with color encoding. Combined with spectral Doppler, further hemodynamic characterization is possible, including waveform morphology and flow velocity measurements [96].

This functionality is especially valuable in pleural procedures, where precise vascular mapping is crucial for safe needle placement and avoiding iatrogenic vascular injury.

### 6.2. Technical Parameters in Ultrasonography: Probe Selection, Machine Configuration, and Scanning Techniques

The selection of an ultrasound probe for thoracic use involves balancing image resolution and tissue penetration [97]. High-frequency transducers provide superior spatial resolution but have limited depth due to higher acoustic attenuation [98]. Linear array probes offer excellent detail for superficial chest wall structures, while low-frequency convex (curvilinear) probes (1–5 MHz) are preferred in patients with increased muscle mass or subcutaneous fat, where deeper penetration is needed [99].

For assessing ICAs, a high-frequency linear probe (5–12 MHz) is generally recommended as the first choice [100]. However, in individuals with substantial subcutaneous tissue, a low-frequency curvilinear probe may be necessary for adequate vessel visualization [12].

**Figure 8:**
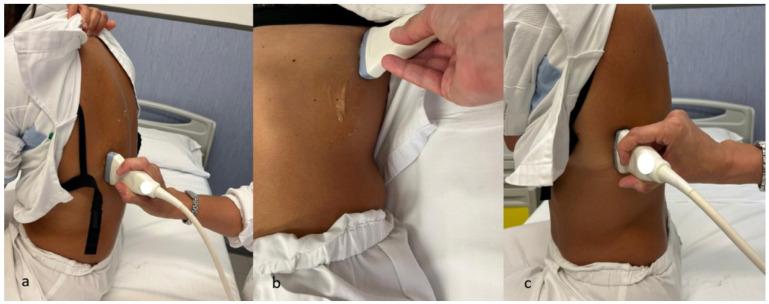
Probe positioning for ultrasound assessment of the thoracic wall: (**a**) Posterior thoracic scan: the patient is seated, and a linear probe is placed perpendicular to the ribs in the posterior thoracic region. (**b**) Anterior thoracic scan: with the patient in the supine position, the linear probe is applied perpendicular to the ribs on the anterior chest wall. (**c**) Lateral thoracic scan: the patient is seated, and the probe is positioned perpendicular to the ribs in the lateral thoracic area.

The scanning technique involves placing the linear probe perpendicular to the ribs at the expected intervention site. Patient positioning varies by anatomical region: supine for anterior chest wall, seated with arm raised for lateral thorax, and contralateral shoulder positioning for subscapular areas [101] (Figure 8).

**Figure 9:**
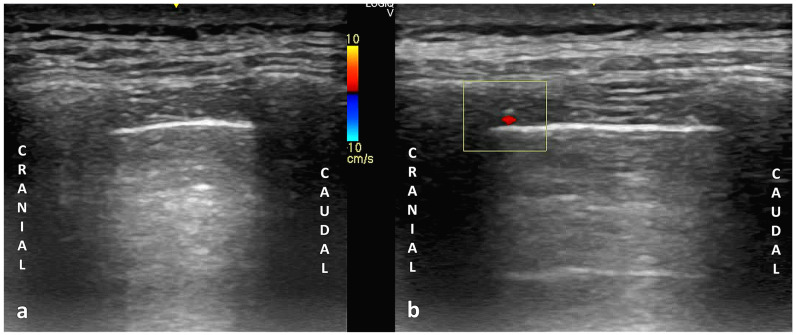
Sonographic visualization of the intercostal artery with a high-frequency linear probe: (**a**) B-mode image of the intercostal space acquired in longitudinal orientation (cranial to the left, caudal to the right). (**b**) Color Doppler image of the same region, showing an arterial Doppler signal (highlighted in the yellow box) within the neurovascular bundle located in the costal groove along the inferior border of the superior rib, consistent with the anatomical course of the intercostal artery.

The intercostal artery typically appears on color and power Doppler as a pulsatile, round structure located along the inferior margin of the superior rib (Figure 9). This anatomical pattern is most common; however, notable variability exists, especially in the elderly [102].

A prospective study of 596 procedures supports using low-frequency curvilinear probes with standardized color Doppler settings at ±18.2 cm·s^−1^. The Doppler ROI should extend from the skin surface to ~2 cm below the parietal pleura, with receiver gain optimized to reduce noise while preserving vessel sensitivity [10].

Another study advocates high-frequency linear probes (8–9 MHz) placed perpendicular to intercostal spaces, with pulse repetition frequencies between 8 and 14 cm/s. A systematic “fanning” motion—sweeping from inferior to superior rib shadow—increases detection of vascular structures [14].

Quality assurance guidelines recommend maintaining Mechanical Index (MI) ≤ 0.4 during chest wall exams, strict adherence to ALARA (As Low As Reasonably Achievable) principles, and continuous MI monitoring throughout scanning, consistent with thoracic ultrasound protocols [103].

## 7. Summary and Analysis of Major Studies Assessing Color Doppler Screening Prior to Pleural Procedures

The use of CDUS to identify the ICA during TUS has emerged as a promising technique to enhance the safety of pleural procedures. Although its impact on reducing bleeding complications and improving clinical outcomes remains to be fully established, several studies have explored the feasibility and diagnostic performance of ICA visualization using Doppler modalities (Table 2).

One of the earliest investigations in this field was conducted by Koyanagi et al. in 2009, who used CDUS to examine intercostal vessels in 12 healthy young male volunteers across various thoracic levels [13]. The study provided important initial insights into the Doppler appearance and flow characteristics of the ICA, laying the groundwork for subsequent work in clinical populations.

A more clinically focused study followed in 2012, when Salamonsen et al. assessed ICA identification in 22 consecutive patients undergoing ultrasound-guided thoracentesis [14]. Across 88 intercostal spaces, the ICA was successfully visualized in 74, with its most frequent location found centrally within the intercostal space—approximately 28% of the intercostal width—compared to only 8% in more axillary regions. These findings supported a segmental anatomical distribution of the ICA and suggested a lateral migration toward the superior rib border in more peripheral zones. The study also corroborated prior anatomical data indicating that the ICA may descend up to 20% into the intercostal space, even at the mid-axillary line [104].

In a subsequent and more comprehensive study, Salamonsen et al. enrolled 50 patients who underwent both TUS and contrast-enhanced CT angiography, the latter serving as the gold standard for ICA localization [105]. Ultrasound examinations were performed by both a pulmonologist and a trained sonographer, using both portable and high-end ultrasound systems. With portable ultrasound, the initial sensitivity for ICA detection was 86%, but specificity was limited to 30%. However, when a modified interpretive criterion was applied—classifying arteries within the superior 15% of the intercostal space as “protected” and thus not at procedural risk—diagnostic performance improved substantially, yielding 95% sensitivity and 97% specificity. The study also reported efficient scan durations: a median of 42 s with portable devices and 18 s with high-end systems. Notably, 77% of scans were conducted by trainees without formal radiology training, demonstrating the feasibility of ICA identification across varying experience levels and without reliance on advanced imaging platforms.

Despite encouraging results, these early studies were constrained by relatively small sample sizes and the absence of a validated, standardized scanning protocol.

A major advancement in the field came with the prospective observational study by Bedawi et al., representing the largest and most comprehensive investigation to date on ICA screening during pleural procedures in real-world clinical practice [10]. Conducted at a single high-volume tertiary center, the study evaluated 596 ultrasound-guided pleural interventions performed by respiratory physicians and trainees without formal radiology or advanced ultrasound training. The operators employed low-frequency curvilinear probes and CDUS, consistent with standard pleural practice, and attempted ICA screening in 95% of procedures, with successful ICA identification achieved in 53% of cases.

The study demonstrated that ICA identification is feasible even without specialist imaging equipment or additional scanning time—typically requiring no more than two minutes to perform. Notably, ICA visualization led to a change in the intended puncture site in nearly one-third of cases and prompted procedural abandonment in 2%, indicating a meaningful impact on safety-oriented decision-making. ICA detection rates were higher in posterior approaches and in cases of pleural thickening, particularly among patients undergoing image-guided biopsy or medical thoracoscopy—likely due to altered anatomical relationships or improved vessel visibility.

Another salient contribution of the study is its demonstration that ICA screening can be reliably performed across a broad range of operator experience levels. Importantly, procedural adjustments were made selectively, even when the ICA was identified, depending on vessel location and trajectory relative to the puncture path—highlighting operator judgment in risk stratification. The study also underscored that advanced transducers were not necessary and that use of the most lateral procedural site—as recommended by pleural guidelines—remains compatible with effective ICA visualization. Altogether, this work supports the integration of ICA screening into routine pleural practice as a pragmatic and generalizable safety step.

A recent contribution to procedural standardization in pleural interventions is the introduction of the DIVOT (Doppler Imaging for Vascular Orientation in Thoracic procedures) protocol, proposed by Fraser et al. [106]. This structured approach outlines a stepwise method for ICA screening using a high-frequency linear probe with color and power Doppler modalities. To minimize the risk of ICA injury, the protocol emphasizes precise transducer orientation, optimal Doppler settings, and a systematic evaluation of the vascular anatomy at the intended puncture site

Recognizing that two-dimensional ultrasound alone cannot reliably visualize vascular structures, the DIVOT protocol provides a practical solution by incorporating flow-sensitive Doppler imaging to identify ICAs and their collateral branches. This is particularly relevant given the known anatomical variability and tortuosity of intercostal vessels, which may lie outside the traditional “safe zone” assumed when inserting a needle over the superior rib margin. Importantly, the DIVOT protocol is adaptable to both seated and supine patient positioning and can be integrated seamlessly into routine pre-procedural scanning. In experienced hands, it adds approximately two minutes to the workflow and requires minimal additional training, suggesting its feasibility for widespread implementation.

While prior studies such as that of Bedawi et al. [10] demonstrated the feasibility of ICA screening using low-frequency curvilinear probes, the DIVOT protocol builds on this by employing higher-resolution imaging and more advanced Doppler settings, potentially enhancing the sensitivity for detecting small or tortuous vessels. Preliminary institutional experience suggests that the protocol is teachable and efficient and may significantly improve safety margins, especially in patients at higher risk of hemorrhagic complications.

While preliminary findings from the aforementioned studies are encouraging, current consensus guidelines continue to advocate for a cautious and measured approach to clinical implementation, and CDUS cannot yet be considered a routine recommendation.

The 2021 European Respiratory Society (ERS) Statement on Thoracic Ultrasound acknowledges that CDUS can be used pre-procedurally to screen for ICAs and post-procedurally to exclude hemorrhage in pleural interventions and image-guided biopsies. However, it emphasizes that the technique remains highly operator- and experience-dependent and that current evidence is insufficient to conclusively establish its diagnostic accuracy or reliability [11].

Similarly, the 2023 British Thoracic Society (BTS) guidelines on pleural procedures acknowledge the potential role of CDUS in identifying intercostal vessels before and after ultrasound-guided interventions, particularly to reduce hemorrhagic complications [107]. While recognizing the technique’s promise, the guidelines underscore the limited evidence supporting its efficacy in bleeding prevention and formally designate this as a key area for future investigation. Accordingly, the accompanying BTS Clinical Statement identifies it as a research priority, explicitly posing the following question: “Does the use of Doppler ultrasound to identify intercostal vessels reduce the risk of puncture and bleeding complications during pleural aspiration (both diagnostic and therapeutic)?” [107].

To the best of our knowledge, the previously cited studies constitute the most substantial contributions published to date regarding the role of CDUS screening prior to pleural procedures. Although the growing body of data supports its feasibility and diagnostic performance, definitive evidence demonstrating that ICA screening effectively reduces procedural complications and improves patient safety is still lacking, and the absence of robust trial data has so far prevented guideline bodies from recommending CDUS as a standard or routine step in pleural interventions. Furthermore, the clinical utility of CDUS must also be interpreted within the context of global healthcare disparities and resource availability. In many low- and middle-income countries, access to advanced ultrasound systems with high-quality Doppler capabilities is limited, and the technique requires not only sophisticated equipment but also specialized training and operator expertise, which may restrict its adoption. In such settings, the cost-effectiveness of implementing CDUS screening protocols must be weighed against alternative safety strategies and competing healthcare priorities.

**Table 2:** Summary of key studies on color Doppler ultrasound for intercostal artery identification during pleural procedures.

Study	Year	Design	Population	Procedure Type	Main Findings	Limitations
Koyanagi et al. [13]	2009	Observational cohort	12 healthy young male volunteers	Transthoracic Doppler sonography	Demonstrated feasibility of visualizing intercostal vessels and assessing arterial flow using Doppler ultrasound in healthy subjects.	Small, homogeneous sample; no procedural outcomes evaluated.
Salamonsen et al. [14]	2012	Prospective cohort	22 patients undergoing US-guided thoracentesis	Thoracentesis	ICA identified in 74/88 intercostal spaces; most frequently located centrally (28% width); notable anatomical variability observed.	Small sample size; absence of standardized protocol; no outcome assessment.
Salamonsen et al. [105]	2013	Prospective cohort	50 patients; LUS + CT angiography	Thoracic ultrasound	Portable US had 86% sensitivity and 30% specificity vs. CT angiography; improved to 95%/97% using a “protected zone” model (ICA in upper 15%); scan time < 1 min; 77% performed by non-radiologists.	No clinical outcome correlation; moderate sample size; limited to one imaging protocol.
Bedawi et al. [10]	2020	Prospective cohort	596 patients undergoing pleural procedures	Thoracentesis, pleural biopsy	ICA screening attempted in 95%, successful in 53%; site changed in 16% overall and 30% when ICA-visualized; low complication rate (0.17%); performed by non-radiologists using low-frequency probes.	Observational design; no direct correlation with bleeding risk; linear probes not assessed.
Fraser et al. [106]	2025	Protocol proposal	Not applicable	Pleural procedures	Introduced the DIVOT protocol for standardized ICA screening using Doppler ultrasound, including patient positioning, probe technique, and safety zones.	No clinical validation; not yet tested in a prospective or multicenter study.

## 8. Limitations, Challenges, and Barriers in the Implementation of Color Doppler Thoracic Ultrasound in Pleural Procedures

Although CDUS has shown promise in identifying ICAs and potentially improving pleural procedure safety, several limitations and barriers hinder its clinical integration. These stem from both methodological weaknesses in the literature and practical challenges across healthcare settings.

The early study by Koyanagi et al. laid important groundwork but was limited by its descriptive design and a homogeneous cohort of healthy young males [13]. The lack of pathological conditions, clinical endpoints, or procedures limits translational value. While it offered insight into thoracic Doppler signatures, it did not assess procedural relevance or safety.

Salamonsen et al. advanced the field with studies in clinical populations, yet both the 2012 and 2013 studies were single-center and small in scale [14,105]. The 2012 study lacked controls, outcome data, and interobserver assessment. The 2013 follow-up added CT angiography and proposed a Doppler-based “safe zone” (superior 15% of the intercostal space), but this threshold remains arbitrary and unvalidated. Variable sensitivity and specificity, absence of bleeding outcomes, and no procedural adaptation limit clinical applicability. While novice operators were included, learning curves and reliability were not evaluated.

More recent work by Bedawi et al. and Fraser et al. highlights persistent issues [10,106]. Single-center designs, equipment variability, and lack of external validation limit generalizability. Bedawi et al. emphasized the trade-off between clinical realism using curvilinear probes and potential under-detection versus linear probes. The assumption that non-visualization equals safety remains unproven [10].

Similarly, the DIVOT protocol by Fraser et al. offers a structured ICA screening approach yet lacks outcome validation and relies heavily on operator skill and high-end equipment [106]. It risks false reassurance in patients with anatomical variants or poor acoustic windows.

Across all studies, a lack of standardized scanning protocols, interpretation criteria, and training introduces heterogeneity that affects image quality and clinical decisions. Moreover, the absence of outcome-based endpoints—such as bleeding, procedural changes, or cost-effectiveness—prevents a full assessment of CDUS impact.

In summary, while CDUS shows feasibility and theoretical benefit for ICA detection, widespread use is still limited by technical, logistical, and evidentiary gaps that preclude consistent adoption in pleural practice.

## 9. Future Perspectives and Research Priorities

The integration of CDUS into routine pleural practice represents a promising but underdeveloped frontier in thoracic procedural safety. Having reached a clear understanding of the current limitations, the path forward must now focus on generating high-quality evidence, developing standardized methodologies, and embracing emerging technologies to optimize its clinical impact.

A central research priority is the conduct of prospective, multicenter studies specifically designed to evaluate whether Doppler-guided vascular screening reduces hemorrhagic complications and improves patient outcomes. Such studies should include stratified cohorts with defined risk profiles—such as patients with coagulopathies, pleural malignancies, or prior thoracic surgery—and assess not only bleeding rates but also changes in procedural planning, need for rescue interventions, and overall cost-effectiveness. The inclusion of well-defined control groups and reproducible ultrasound protocols will be essential to ensure scientific rigor and clinical applicability.

Equally important is the establishment of standardized scanning techniques and interpretive criteria that can be widely adopted across centers, independent of operator experience or equipment variability. Consensus-driven protocols—potentially modeled on frameworks like the DIVOT protocol—represent promising initial approaches but require extensive validation in larger, multicenter, outcome-based studies before they can be reliably incorporated into international guidelines for pleural procedures.

Hybrid imaging strategies also merit exploration. The integration of CDUS with contrast-enhanced CT or MRI may be particularly useful in anatomically complex or high-risk patients [108], such as those with distorted pleural anatomy or prior surgical adhesions. The development of fusion imaging platforms, capable of overlaying vascular maps from CT onto real-time ultrasound, could offer a novel solution to procedural planning challenges in selected cases [109,110,111,112,113].

In parallel, attention should be directed toward pleural scenarios where Doppler imaging may have specific value—such as image-guided biopsies in hypervascular tumors or repeat procedures in those with prior bleeding complications [114,115]. The combination of color Doppler with contrast-enhanced ultrasound may further enable detailed vascular characterization of pleural surfaces, enhancing procedural precision [116,117].

Artificial intelligence (AI) applications show preliminary promise in the management and follow-up of pleural diseases; however, their clinical use remains largely investigational [118,119,120,121,122,123]. Although early studies suggest potential benefits for diagnostic accuracy and procedural safety, robust clinical validation is still lacking.

The potential application of AI in Doppler ultrasound represents an area of active research interest. Deep learning algorithms are being explored for real-time vessel recognition, flow quantification, and automated flagging of high-risk vascular trajectories [124,125,126,127,128,129]. However, these technologies face significant challenges including the need for extensive training datasets, validation across diverse patient populations and equipment platforms, and demonstration of clinical superiority over current methods.

While such AI-driven tools could theoretically reduce operator dependency, improve reproducibility, and accelerate training, several critical limitations must be addressed: current algorithms require substantial computational resources, may not perform consistently across different ultrasound systems, and lack the extensive clinical validation necessary for routine implementation. Furthermore, the integration of AI into clinical workflows presents practical challenges regarding cost-effectiveness and user acceptance.

Although these innovations may eventually improve access to advanced ultrasound techniques and enhance procedural safety, their clinical impact remains unproven. Substantial research, rigorous validation studies, and careful evaluation of real-world effectiveness will be essential before these technologies can be considered for routine clinical implementation in pleural interventions.

## 10. Conclusions

Hemorrhagic complications from pleural interventions, though infrequent, represent a critical safety concern due to their potentially life-threatening consequences. Despite advances in ultrasound guidance and procedural technique, ICA injury remains a notable risk that is not fully mitigated by current practice standards. CDUS has demonstrated feasibility and diagnostic potential for pre-procedural ICA identification across diverse operator experience levels and clinical settings. However, widespread adoption is limited by heterogeneity in scanning protocols, operator dependency, and insufficient prospective outcome data.

The expanding complexity and volume of pleural procedures, alongside the evolving landscape of pleural medicine, underscore the urgent need for standardized ICA screening protocols supported by robust, multicenter clinical trials. Integration of advanced imaging modalities and hybrid techniques may further refine vascular mapping, particularly in high-risk populations. Moreover, emerging AI applications offer a promising avenue to enhance real-time vessel recognition, reduce operator variability, and improve procedural safety and efficiency.

Ultimately, ensuring patient safety demands a coordinated approach encompassing rigorous research, comprehensive training, and incorporation of innovative technologies. While Doppler-guided ICA screening is not yet the standard of care, it holds considerable promise as a critical component of pleural procedural planning. Future efforts must focus on generating definitive evidence to inform guidelines and facilitate widespread, evidence-based implementation, with the overarching goal of minimizing preventable vascular complications and optimizing outcomes in pleural medicine. Patient safety remains at the core of our practice, and every effort should be made to enhance this fundamental principle.

## Figures and Tables

**Table 1:** Summary of anatomical variations reported in cadaveric dissections and virtual 3D models from various studies. These data provide important insights into human vascular anatomy and its variability. While virtual 3D models are increasingly utilized for educational purposes, autopsy studies remain crucial for accurate anatomical characterization and surgical planning.

Authors	Year	Cadaveric Specimens (*n*)	Age (Mean)	Observed Anatomical Variations in PIAs
Nathan et al. [25]	1970	50	NA	Location of the orifices Course variations
Khan and Haust [26]	1979	79	7.5 years	Size and location of the orifices Numerosity variations (common trunks) Complete or incomplete division of a single artery beyond its origin
Porto da Rocha et al. [27]	2002	90	40 years	Origin, size, and topographic relationships of the collateral intercostal arteries and of the posterior intercostal arteries
Shimizu et al. [28]	2005	5	NA	Location of the orifices
Kocbek et al. [29]	2011	44	NA	Origin and course
Shurtleff and Olinger [30]	2012	29	71 years	Tortuosity Point of origin of collateral branches Dimension of collateral branches
Kocbek and Rakuša [31]	2018	44	NA	Numerosity variations (common trunks) and their prevalence
Kocbek Šaherl et al. [32]	2020	43	NA	Density, position, and networking of collateral branches

## Data Availability

Not applicable. No new data were created or analyzed in this study.

## Abbreviations

The following abbreviations are used in this manuscript:

ACCP	American College of Chest Physicians
AI	Artificial Intelligence
AIA	Anterior Intercostal Artery
ALARA	As Low As Reasonably Achievable
ATS	American Thoracic Society
BTS	British Thoracic Society
CDUS	Color Doppler Thoracic Ultrasound
CECT	Contrast-Enhanced Computed Tomography
CTA	CT Angiography
DIC	Disseminated Intravascular Coagulation
DIVOT	Doppler Imaging for Vascular Orientation in Thoracic Procedures
DOACs	Direct Oral Anticoagulants
DSA	Digital Subtraction Angiography
ERS	European Respiratory Society
Fr	French (catheter size)
ICA	Intercostal Artery
ICD	Intercostal Chest Drain
INR	International Normalized Ratio
IPC	Indwelling Pleural Catheter
ITA	Internal Thoracic Artery
LUS	Lung Ultrasound
MHz	Megahertz
MI	Mechanical Index
MRI	Magnetic Resonance Imaging
PD	Power Doppler
PE	Pleural Effusion
PIA	Posterior Intercostal Artery
ROI	Region of Interest
TUS	Thoracic Ultrasound
US	Ultrasound
VATS	Video-Assisted Thoracic Surgery

## References

[1] Carlucci P., Trigiani M., Mori P.A., Mondoni M., Pinelli V., Casalini A.G., Conte E.G., Buggio G., Villari L., Marchetti G. (2019). Competence in pleural procedures. Panminerva Med..

[2] Tinè M., Daverio M., Semenzato U., Cocconcelli E., Bernardinello N., Damin M., Saetta M., Spagnolo P., Balestro E. (2023). Pleural clinic: Where thoracic ultrasound meets respiratory medicine. Front. Med..

[3] Psallidas I., Rahman N.M. (2016). Advances in pleural disease. Eur. Respir. Rev..

[4] Evison M., Blyth K.G., Bhatnagar R., Corcoran J., Saba T., Duncan T., Hallifax R., Ahmed L., West A., Pepperell J.C.T. (2018). Providing safe and effective pleural medicine services in the UK: An aspirational statement from UK pleural physicians. BMJ Open Respir. Res..

[5] Corcoran J.P., Psallidas I., Wrightson J.M., Hallifax R.J., Rahman N.M. (2015). Pleural procedural complications: Prevention and management. J. Thorac. Dis..

[6] Dunscombe A.O., Maskell N.A. (2012). Common iatrogenic pleural complications. Curr. Respir. Care Rep..

[7] Laugsand E.A., Xanthoulis A. (2020). Management of a life-threatening intercostal artery bleeding, difficult to visualize in open surgery: A case report. J. Surg. Case Rep..

[8] Psallidas I., Helm E.J., Maskell N.A., Yarmus L., Feller-Kopman D.J., Gleeson F.V., Rahman N.M. (2015). Iatrogenic injury to the intercostal artery: Aetiology, diagnosis and therapeutic intervention. Thorax.

[9] Cantey E.P., Walter J.M., Corbridge T., Barsuk J.H. (2016). Complications of thoracentesis: Incidence, risk factors, and strategies for prevention. Curr. Opin. Pulm. Med..

[10] Bedawi E.O., Talwar A., Hassan M., McCracken D.J., Asciak R., Mercer R.M., Kanellakis N.I., Gleeson F.V., Hallifax R.J., Wrightson J.M. (2020). Intercostal vessel screening prior to pleural interventions by the respiratory physician: A prospective study of real world practice. Eur. Respir. J..

[11] Laursen C.B., Clive A., Hallifax R., Pietersen P.I., Asciak R., Davidsen J.R., Bhatnagar R., Bedawi E.O., Jacobsen N., Coleman C. (2021). European Respiratory Society statement on thoracic ultrasound. Eur. Respir. J..

[12] Kanai M., Sekiguchi H. (2015). Avoiding vessel laceration in thoracentesis: A role of vascular ultrasound with color Doppler. Chest.

[13] Koyanagi T., Kawaharada N., Kurimoto Y., Ito T., Baba T., Nakamura M., Watanebe A., Higami T. (2010). Examination of intercostal arteries with transthoracic Doppler sonography. Echocardiography.

[14] Salamonsen M., Ellis S., Paul E., Steinke K., Fielding D. (2012). Thoracic ultrasound demonstrates variable location of the intercostal artery. Respiration.

[15] Roberts M.E., Rahman N.M., Maskell N.A., Bibby A.C., Blyth K.G., Corcoran J.P., Edey A., Evison M., de Fonseka D., Hallifax R. (2023). British Thoracic Society Guideline for pleural disease. Thorax.

[16] Gray H., Standring S., Anand N., Tunstall R. (2021). Gray’s Anatomy: The Anatomical Basis of Clinical Practice.

[17] Donley E.R., Holme M.R., Loyd J.W. (2025). Anatomy, Thorax, Wall Movements. StatPearls.

[18] Dewhurst C., O’Neill S., O’Regan K., Maher M. (2012). Demonstration of the course of the posterior intercostal artery on CT angiography: Relevance to interventional radiology procedures in the chest. Diagn. Interv. Radiol..

[19] Granger C.J., Martin A.R. (2025). Anatomy, Thorax, Superior Intercostal Arteries. StatPearls.

[20] Elsy B. (2025). Clinically relevant anatomical variations in the posterior intercostal neurovascular bundle. Folia Morphol..

[21] Glenesk N.L., Rahman S., Lopez P.P. (2025). Anatomy, Thorax, Intercostal Nerves. StatPearls.

[22] Hawi J.S., Jurjus R.A., Daouk H.S., Ghazi M.N., Basset C.A., Cappello F., Hajj Hussein I., Leone A., Jurjus A.R. (2023). A Rare Bilateral Variation in the Branches of the Internal Thoracic Artery: A Case Report. Anatomia.

[23] Agnihotri G., Mitra A. (2022). A study on origin, termination, and course characteristics of internal thoracic artery relevant to coronary surgeries and reconstructive procedures. Tzu Chi Med. J..

[24] Rakuša M., Kocbek Šaherl L. (2023). Thiel embalming method used for anatomy dissection as an educational tool in teaching human anatomy, in research, and in training in comparison of different methods for long term preservation. Folia Morphol..

[25] Nathan H., Barkay M., Orda R. (1970). Anatomical observations on the origin and course of the aortic intercostal arteries. J. Thorac. Cardiovasc. Surg..

[26] Khan S., Haust M.D. (1979). Variations in the aortic origin of intercostal arteries in man. Anat. Rec..

[27] Da Rocha R.P., Vengjer A., Blanco A., de Carvalho P.T., Mongon M.L.D., Fernandes G.J.M. (2002). Size of the collateral intercostal artery in adults: Anatomical considerations in relation to thoracocentesis and thoracoscopy. Surg. Radiol. Anat..

[28] Shimizu S., Tanaka R., Kan S., Suzuki S., Kurata A., Fujii K. (2005). Origins of the segmental arteries in the aorta: An anatomic study for selective catheterization with spinal arteriography. AJNR Am. J. Neuroradiol..

[29] Kocbek L., Krajnc I., Anderhuber F. (2011). Anatomical variations of the posterior intercostal arteries and the thoracic vertebral artery. J. Int. Med. Res..

[30] Shurtleff E., Olinger A. (2012). Posterior intercostal artery tortuosity and collateral branch points: A cadaveric study. Folia Morphol..

[31] Kocbek L., Rakuša M. (2018). Common trunk of the posterior intercostal arteries from the thoracic aorta: Anatomical variation, frequency, and importance in individuals. Surg. Radiol. Anat..

[32] Šaherl L.K., Gosak M., Rakuša M. (2020). Identification and quantitative analysis of branching networks of the posterior intercostal arteries. Anat. Sci. Int..

[33] Fanselow N.R., Wallace N., Sehi D., Coomar L., Martin J., Tan Y., Daly D.T. (2021). A Case of Multiple Posterior Intercostal Artery Common Trunks in Conjunction with Additional Arterial Variations. Case Rep. Surg..

[34] Choi S., Trieu J., Ridley L. (2010). Radiological review of intercostal artery: Anatomical considerations when performing procedures via intercostal space. J. Med. Imaging Radiat. Oncol..

[35] Helm E.J., Rahman N.M., Talakoub O., Fox D.L., Gleeson F.V. (2013). Course and variation of the intercostal artery by CT scan. Chest.

[36] Yoshioka K., Niinuma H., Ehara S., Nakajima T., Nakamura M., Kawazoe K. (2006). MR angiography and CT angiography of the artery of Adamkiewicz: State of the art. Radiographics.

[37] Maskell N.A., Laursen C.B., Lee Y.C.G. (2020). Pleural Disease.

[38] Light R.W. (2007). Pleural diseases.

[39] Feller-Kopman D., Light R. (2018). Pleural Disease. N. Engl. J. Med..

[40] Vakil E., Taghizadeh N., Tremblay A. (2023). The Global Burden of Pleural Diseases. Semin. Respir. Crit. Care Med..

[41] Mummadi S.R., Stoller J.K., Lopez R., Kailasam K., Gillespie C.T., Hahn P.Y. (2021). Epidemiology of Adult Pleural Disease in the United States. Chest.

[42] Bodtger U., Hallifax R.J., Maskell N.A., Laursen C.B., Lee Y.C.G., Rahman N.M. (2020). Epidemiology: Why is pleural disease becoming more common?. Pleural Disease.

[43] Nicholson M.J., Manley C., Ahmad D. (2023). Thoracentesis for the Diagnosis and Management of Pleural Effusions: The Current State of a Centuries-Old Procedure. J. Respir..

[44] Ferreiro L., Suárez-Antelo J., Toubes M.E., Valdés L. (2019). Thoracentesis in Primary Care. Semergen.

[45] Shen-Wagner J., Gamble C., MacGilvray P. (2023). Pleural Effusion: Diagnostic Approach in Adults. Am. Fam. Physician.

[46] Sorino C., Feller-Kopman D., Mei F., Mondoni M., Agati S., Marchetti G., Rahman N.M. (2024). Chest Tubes and Pleural Drainage: History and Current Status in Pleural Disease Management. J. Clin. Med..

[47] Anderson D., Chen S.A., Godoy L.A., Brown L.M., Cooke D.T. (2022). Comprehensive Review of Chest Tube Management: A Review. JAMA Surg..

[48] Porcel J.M. (2018). Chest Tube Drainage of the Pleural Space: A Concise Review for Pulmonologists. Tuberc. Respir. Dis..

[49] Mei F., Bonifazi M., Rota M., Cirilli L., Grilli M., Duranti C., Zuccatosta L., Bedawi E.O., McCracken D., Gasparini S. (2021). Diagnostic Yield and Safety of Image-Guided Pleural Biopsy: A Systematic Review and Meta-Analysis. Respiration.

[50] Benamore R.E., Scott K., Richards C.J., Entwisle J.J. (2006). Image-guided pleural biopsy: Diagnostic yield and complications. Clin. Radiol..

[51] Cagle P.T., Allen T.C. (2011). Pathology of the pleura: What the pulmonologists need to know. Respirology.

[52] Boy D. (2023). Ultrasound-guided pleural biopsy. Eurasian J. Pulmonol..

[53] Mondoni M., Saderi L., Trogu F., Terraneo S., Carlucci P., Ghelma F., Centanni S., Sotgiu G. (2021). Medical thoracoscopy treatment for pleural infections: A systematic review and meta-analysis. BMC Pulm. Med..

[54] Pinelli V., Clive A.O., Maskell N.A., Laursen C.B., Lee Y.C.G., Rahman N.M. (2020). Medical thoracoscopy in 2020: Essential and future techniques. Pleural Disease.

[55] Avasarala S.K., Lentz R.J., Maldonado F. (2021). Medical Thoracoscopy. Clin. Chest Med..

[56] Bhatnagar R., Corcoran J.P., Maldonado F., Feller-Kopman D., Janssen J., Astoul P., Rahman N.M. (2016). Advanced medical interventions in pleural disease. Eur. Respir. Rev..

[57] Luzzi V., Lindahl A.L., Tomassetti S. (2025). Indwelling pleural catheters and medical thoracoscopy. Breathe.

[58] Marchi G., Cucchiara F., Gregori A., Biondi G., Guglielmi G., Serradori M., Gherardi M., Gabbrielli L., Pistelli F., Carrozzi L. (2025). Thoracic Ultrasound for Pre-Procedural Dynamic Assessment of Non-Expandable Lung: A Non-Invasive, Real-Time and Multifaceted Diagnostic Tool. J. Clin. Med..

[59] Marchi G. (2025). The road less travelled: Thoracic ultrasound, advanced imaging, and artificial intelligence for early diagnosis of non-expandable lung in malignant pleural effusion. Breathe.

[60] Baguneid A., Wijayaratne T., Aujayeb A., Panchal R. (2025). The Evolution of the Indwelling Pleural Catheter. Pulm. Ther..

[61] Castaldo N., Fantin A., Palou-schwartzbaum M., Viterale G., Crisafulli E., Sartori G., Aujayeb A., Patrucco F., Patruno V. (2024). Exploring the efficacy and advancements of medical pleurodesis: A comprehensive review of current research. Breathe.

[62] Mierzejewski M., Korczynski P., Krenke R., Janssen J.P. (2019). Chemical pleurodesis—A review of mechanisms involved in pleural space obliteration. Respir. Res..

[63] Bhatnagar R., Piotrowska H.E.G., Laskawiec-Szkonter M., Kahan B.C., Luengo-Fernandez R., Pepperell J.C.T., Evison M.D., Holme J., Al-Aloul M., Psallidas I. (2020). Effect of Thoracoscopic Talc Poudrage vs Talc Slurry via Chest Tube on Pleurodesis Failure Rate Among Patients with Malignant Pleural Effusions: A Randomized Clinical Trial. JAMA.

[64] Martinez-Zayas G., Molina S., Ost D.E. (2022). Sensitivity and complications of thoracentesis and thoracoscopy: A meta-analysis. Eur. Respir. Rev..

[65] Sundaralingam A., Bedawi E.O., Harriss E.K., Munavvar M., Rahman N.M. (2022). The Frequency, Risk Factors, and Management of Complications from Pleural Procedures. Chest.

[66] Hernandez M.C., El Khatib M., Prokop L., Zielinski M.D., Aho J.M. (2018). Complications in tube thoracostomy: Systematic review and meta-analysis. J. Trauma Acute Care Surg..

[67] Williams J.G., Lerner A.D. (2021). Managing complications of pleural procedures. J. Thorac. Dis..

[68] Iyer N.P., Reddy C.B., Wahidi M.M., Lewis S.Z., Diekemper R.L., Feller-Kopman D., Gould M.K., Balekian A.A. (2019). Indwelling Pleural Catheter versus Pleurodesis for Malignant Pleural Effusions. A Systematic Review and Meta-Analysis. Ann. Am. Thorac. Soc..

[69] Bedawi E.O., Ricciardi S., Hassan M., Gooseman M.R., Asciak R., Castro-Añón O., Armbruster K., Bonifazi M., Poole S., Harris E.K. (2023). ERS/ESTS statement on the management of pleural infection in adults. Eur. Respir. J..

[70] Zeiler J., Idell S., Norwood S., Cook A. (2020). Hemothorax: A Review of the Literature. Clin. Pulm. Med..

[71] Azfar Ali H., Lippmann M., Mundathaje U., Khaleeq G. (2008). Spontaneous Hemothorax. Chest.

[72] Mahmood K., Shofer S.L., Moser B.K., Argento A.C., Smathers E.C., Wahidi M.M. (2014). Hemorrhagic complications of thoracentesis and small-bore chest tube placement in patients taking clopidogrel. Ann. Am. Thorac. Soc..

[73] DiVietro M.L., Huggins J.T., Angotti L.B., Kummerfeldt C.E., Nestor J.E., Doelken P., Sahn S.A. (2015). Pleural Fluid Analysis in Chronic Hemothorax: A Mimicker of Infection. Clin. Med. Insights Case Rep..

[74] Ault M.J., Rosen B.T., Scher J., Feinglass J., Barsuk J.H. (2015). Thoracentesis outcomes: A 12-year experience. Thorax.

[75] Puchalski J.T., Argento A.C., Murphy T.E., Araujo K.L.B., Pisani M.A. (2013). The safety of thoracentesis in patients with uncorrected bleeding risk. Ann. Am. Thorac. Soc..

[76] Aljundi L., Chaar A., Boshara P., Shiari A., Gennaoui G., Noori Z., Girard C., Szpunar S., Franco-Elizondo R. (2021). Incidence of bleeding in patients on different anticoagulants and antiplatelet therapies undergoing thoracentesis. BMJ Open Respir. Res..

[77] Giri M., Dai H., Guo S., Li Y., He L., Zhuang R. (2022). Efficacy and Safety of Pleural Cryobiopsy vs. Forceps Biopsy for Evaluation of Undiagnosed Pleural Effusion: A Systematic Review and Meta-Analysis. Front. Med..

[78] Rahman N.M., Ali N.J., Brown G., Chapman S.J., Davies R.J.O., Downer N.J., Gleeson F.V., Howes T.Q., Treasure T., Singh S. (2010). Local anaesthetic thoracoscopy: British Thoracic Society Pleural Disease Guideline 2010. Thorax.

[79] Mowery N.T., Gunter O.L., Collier B.R., Diaz J.J., Haut E., Hildreth A., Holevar M., Mayberry J., Streib E. (2011). Practice management guidelines for management of hemothorax and occult pneumothorax. J. Trauma.

[80] Meyer D.M., Jessen M.E., Wait M.A., Estrera A.S. (1997). Early evacuation of traumatic retained hemothoraces using thoracoscopy: A prospective, randomized trial. Ann. Thorac. Surg..

[81] Velmahos G.C., Demetriades D., Chan L., Tatevossian R., Cornwell E.E., Yassa N., Murray J.A., Asensio J.A., Berne T.V. (1999). Predicting the need for thoracoscopic evacuation of residual traumatic hemothorax: Chest radiograph is insufficient. J. Trauma.

[82] DuBose J., Inaba K., Demetriades D., Scalea T.M., O’Connor J., Menaker J., Morales C., Konstantinidis A., Shiflett A., Copwood B. (2012). Management of post-traumatic retained hemothorax: A prospective, observational, multicenter AAST study. J. Trauma Acute Care Surg..

[83] Oğuzkaya F., Akçali Y., Bilgin M. (2005). Videothoracoscopy versus intrapleural streptokinase for management of post traumatic retained haemothorax: A retrospective study of 65 cases. Injury.

[84] Skeete D.A., Rutherford E.J., Schlidt S.A., Abrams J.E., Parker L.A., Rich P.B. (2004). Intrapleural tissue plasminogen activator for complicated pleural effusions. J. Trauma.

[85] Stiles P.J., Drake R.M., Helmer S.D., Bjordahl P.M., Haan J.M. (2014). Evaluation of chest tube administration of tissue plasminogen activator to treat retained hemothorax. Am. J. Surg..

[86] Feller-Kopman D.J., Reddy C.B., DeCamp M.M., Diekemper R.L., Gould M.K., Henry T., Iyer N.P., Lee Y.C.G., Lewis S.Z., Maskell N.A. (2018). Management of Malignant Pleural Effusions. An Official ATS/STS/STR Clinical Practice Guideline. Am. J. Respir. Crit. Care Med..

[87] Simoff M.J., Lally B., Slade M.G., Goldberg W.G., Lee P., Michaud G.C., Wahidi M.M., Chawla M. (2013). Symptom management in patients with lung cancer: Diagnosis and management of lung cancer, 3rd ed: American College of Chest Physicians evidence-based clinical practice guidelines. Chest.

[88] Hibbert R.M., Atwell T.D., Lekah A., Patel M.D., Carter R.E., McDonald J.S., Rabatin J.T. (2013). Safety of ultrasound-guided thoracentesis in patients with abnormal preprocedural coagulation parameters. Chest.

[89] Keeling D., Tait R.C., Watson H. (2016). British Committee of Standards for Haematology Peri-operative management of anticoagulation and antiplatelet therapy. Br. J. Haematol..

[90] Linder K., Epelbaum O. (2018). Percutaneous pleural drainage in patients taking clopidogrel: Real danger or phantom fear?. J. Thorac. Dis..

[91] Evans D.H., Jensen J.A., Nielsen M.B. (2011). Ultrasonic colour Doppler imaging. Interface Focus.

[92] Carroll D., Knipe H., Bickle I. (2019). Color Flow Doppler (Ultrasound). https://radiopaedia.org/articles/67339.

[93] Magee P. (2020). Essential notes on the physics of Doppler ultrasound. BJA Educ..

[94] Carroll D., Chieng R., Howden W. (2019). Spectral Doppler (Ultrasound). https://radiopaedia.org/articles/67204.

[95] Kitamura F., Agolah D., Haouimi A. (2014). Power Doppler. https://radiopaedia.org/articles/30430.

[96] Görg C., Bert T., Görg K., Heinzel-Gutenbrunner M. (2005). Colour Doppler ultrasound mapping of chest wall lesions. Br. J. Radiol..

[97] Williamson J.P., Grainge C., Parameswaran A., Twaddell S.H. (2017). Thoracic Ultrasound: What Non-radiologists Need to Know. Curr. Pulmonol. Rep..

[98] Nathani A., Keshishyan S., Cho R.J. (2024). Advancements in Interventional Pulmonology: Harnessing Ultrasound Techniques for Precision Diagnosis and Treatment. Diagnostics.

[99] Corcoran J.P., Hew M., Maldonado F., Koegelenberg C.F.N., Laursen C.B., Rahman N.M., Volpicelli G. (2018). Ultrasound-guided procedures. Thoracic Ultrasound.

[100] Colares P.d.F.B., Mafort T.T., Sanches F.M., Monnerat L.B., Menegozzo C.A.M., Mariani A.W. (2024). Thoracic ultrasound: A review of the state-of-the-art. J. Bras. Pneumol..

[101] Bartolomé-Solanas A., Porta-Vilaró M., Soler-Perromat J.C., Del Amo M., García-Diez A.I., Radalov I., Cornellas L., Pomés Lopez I., Isern-Kebschull J., Tomás X. (2024). Narrative review of chest wall ultrasound: A practical approach. Quant. Imaging Med. Surg..

[102] Yoneyama H., Arahata M., Temaru R., Ishizaka S., Minami S. (2010). Evaluation of the risk of intercostal artery laceration during thoracentesis in elderly patients by using 3D-CT angiography. Intern. Med..

[103] Demi L., Wolfram F., Klersy C., De Silvestri A., Ferretti V.V., Muller M., Miller D., Feletti F., Wełnicki M., Buda N. (2023). New International Guidelines and Consensus on the Use of Lung Ultrasound. J. Ultrasound Med..

[104] Wraight W.M., Tweedie D.J., Parkin I.G. (2005). Neurovascular anatomy and variation in the fourth, fifth, and sixth intercostal spaces in the mid-axillary line: A cadaveric study in respect of chest drain insertion. Clin. Anat..

[105] Salamonsen M., Dobeli K., McGrath D., Readdy C., Ware R., Steinke K., Fielding D. (2013). Physician-performed ultrasound can accurately screen for a vulnerable intercostal artery prior to chest drainage procedures. Respirology.

[106] Fraser A., Brenner D.S., Coghlan M., Andrade H., Haouili M., Carlos W.G., Jackson E. (2025). The Sound of Safety: DIVOT (Doppler Imaging for Vascular Orientation in Thoracic Procedures) Protocol. POCUS J..

[107] Asciak R., Bedawi E.O., Bhatnagar R., Clive A.O., Hassan M., Lloyd H., Reddy R., Roberts H., Rahman N.M. (2023). British Thoracic Society Clinical Statement on pleural procedures. Thorax.

[108] Kloth C., Kratzer W., Schmidberger J., Beer M., Clevert D.A., Graeter T. (2021). Ultrasound 2020—Diagnostics & Therapy: On the Way to Multimodal Ultrasound: Contrast-Enhanced Ultrasound (CEUS), Microvascular Doppler Techniques, Fusion Imaging, Sonoelastography, Interventional Sonography. Rofo.

[109] Ewertsen C., Săftoiu A., Gruionu L.G., Karstrup S., Nielsen M.B. (2013). Real-Time Image Fusion Involving Diagnostic Ultrasound. Am. J. Roentgenol..

[110] Bazot M., Spagnoli F., Guerriero S. (2022). Magnetic resonance imaging and ultrasound fusion technique in gynecology. Ultrasound Obstet. Gyne..

[111] Gross J.S., Yaeger A., Tchelepi H., Matcuk G.R. (2023). Ultrasound Fusion: Applications in Musculoskeletal Imaging. Life.

[112] Sakakibara J., Nagashima T., Fujimoto H., Takada M., Ohtsuka M. (2023). A review of MRI (CT)/US fusion imaging in treatment of breast cancer. J. Med. Ultrason. (2001).

[113] Piazza M., Colacchio E.C., Bilato M.J., Squizzato F., Antonello M. (2025). Combining fusion-imaging and intravascular ultrasound guidance to facilitate transcatheter electrosurgical septostomy through preexisting entry tears during endovascular repair of dissecting aneurysms. J. Vasc. Surg. Cases Innov. Tech..

[114] Safai Zadeh E., Weide J., Dietrich C.F., Trenker C., Koczulla A.R., Görg C. (2021). Diagnostic Accuracy of B-Mode- and Contrast-Enhanced Ultrasound in Differentiating Malignant from Benign Pleural Effusions. Diagnostics.

[115] Safai Zadeh E., Görg C., Dietrich C.F., Görlach J., Alhyari A., Trenker C. (2022). Contrast-Enhanced Ultrasound for Evaluation of Pleural Effusion: A Pictorial Essay. J. Ultrasound Med..

[116] Mehta K.S., Lee J.J., Taha A.A., Avgerinos E., Chaer R.A. (2017). Vascular applications of contrast-enhanced ultrasound imaging. J. Vasc. Surg..

[117] Abou Ali A.N., Fittipaldi A., Rocha-Neves J., Ruaro B., Benedetto F., Al Ghadban Z., Simon G., Lepidi S., D’Oria M. (2025). Clinical applications of contrast-enhanced ultrasound in vascular surgery: State-of-the-art narrative and pictorial review. JVS-Vasc. Insights.

[118] Marchi G., Mercier M., Cefalo J., Salerni C., Ferioli M., Candoli P., Gori L., Cucchiara F., Cenerini G., Guglielmi G. (2025). Advanced imaging techniques and artificial intelligence in pleural diseases: A narrative review. Eur. Respir. Rev..

[119] Addala D.N., Rahman N.M. (2024). Man versus Machine in Pleural Diagnostics: Does Artificial Intelligence Provide the Solution?. Ann. ATS.

[120] Kim N.Y., Jang B., Gu K.-M., Park Y.S., Kim Y.-G., Cho J. (2024). Differential Diagnosis of Pleural Effusion Using Machine Learning. Ann. ATS.

[121] Chen Q.-Y., Yin S.-M., Shao M.-M., Yi F.-S., Shi H.-Z. (2025). Machine learning-based Diagnostic model for determining the etiology of pleural effusion using Age, ADA and LDH. Respir. Res..

[122] Causio F.A., DE Angelis L., Diedenhofen G., Talio A., Baglivo F. (2024). Workshop Participants Perspectives on AI use in medicine: Views of the Italian Society of Artificial Intelligence in Medicine. J. Prev. Med. Hyg..

[123] Ishiwata T., Yasufuku K. (2024). Artificial intelligence in interventional pulmonology. Curr. Opin. Pulm. Med..

[124] Jiang B., Chen A., Bharat S., Zheng M. (2021). Automatic ultrasound vessel segmentation with deep spatiotemporal context learning. arXiv.

[125] Nimmagadda N., Aboian E., Kiang S., Fischer U. (2025). The role of artificial intelligence in vascular care. JVS-Vasc. Insights.

[126] Alsharqi M., Edelman E.R. (2025). Artificial Intelligence in Cardiovascular Imaging and Interventional Cardiology: Emerging Trends and Clinical Implications. J. Soc. Cardiovasc. Angiogr. Interv..

[127] Föllmer B., Williams M.C., Dey D., Arbab-Zadeh A., Maurovich-Horvat P., Volleberg R.H.J.A., Rueckert D., Schnabel J.A., Newby D.E., Dweck M.R. (2024). Roadmap on the use of artificial intelligence for imaging of vulnerable atherosclerotic plaque in coronary arteries. Nat. Rev. Cardiol..

[128] Barash Y., Livne A., Klang E., Sorin V., Cohen I., Khaitovich B., Raskin D. (2024). Artificial Intelligence for Identification of Images with Active Bleeding in Mesenteric and Celiac Arteries Angiography. Cardiovasc. Intervent Radiol..

[129] Tran Z., Wilkinson M.C., Guardamondo G., El-Farra M.H., Peetz A.B., Tomihama R.T., Kiang S.C. (2025). Narrative review on applications of artificial intelligence in vascular trauma. JVS-Vasc. Insights.

